# Gyrification, cortical and subcortical morphometry in neurofibromatosis type 1: an uneven profile of developmental abnormalities

**DOI:** 10.1186/1866-1955-5-3

**Published:** 2013-02-13

**Authors:** Inês R Violante, Maria J Ribeiro, Eduardo D Silva, Miguel Castelo-Branco

**Affiliations:** 1 Institute for Biomedical Imaging and Life Sciences, Faculty of Medicine, University of Coimbra, Azinhaga de Santa Comba, Coimbra 3000-548, Portugal; 2 Department of Ophthalmology, Centro Hospitalar e Universitário de Coimbra, EPE, Av. Bissaya Barreto, Coimbra, 3000-075, Portugal

**Keywords:** Brain structure, Gyrification, Morphometry, MRI, Neurodevelopmental disorders, Neurofibromatosis type 1

## Abstract

**Background:**

Neurofibromatosis type 1 (NF1) is a monogenic disorder associated with cognitive impairments. In order to understand how mutations in the *NF1* gene impact brain structure it is essential to characterize in detail the brain structural abnormalities in patients with NF1. Previous studies have reported contradictory findings and have focused only on volumetric measurements. Here, we investigated the volumes of subcortical structures and the composite dimensions of the cortex through analysis of cortical volume, cortical thickness, cortical surface area and gyrification.

**Methods:**

We studied 14 children with NF1 and 14 typically developing children matched for age, gender, IQ and right/left-handedness. Regional subcortical volumes and cortical gyral measurements were obtained using the FreeSurfer software. Between-group differences were evaluated while controlling for the increase in total intracranial volume observed in NF1.

**Results:**

Subcortical analysis revealed disproportionately larger thalami, right caudate and middle corpus callosum in patients with NF1. Cortical analyses on volume, thickness and surface area were however not indicative of significant alterations in patients. Interestingly, patients with NF1 had significantly lower gyrification indices than typically developing children primarily in the frontal and temporal lobes, but also affecting the insula, cingulate cortex, parietal and occipital regions.

**Conclusions:**

The neuroanatomic abnormalities observed were localized to specific brain regions, indicating that particular areas might constitute selective targets for *NF1* gene mutations. Furthermore, the lower gyrification indices were accompanied by a disproportionate increase in brain size without the corresponding increase in folding in patients with NF1. Taken together these findings suggest that specific neurodevelopmental processes, such as gyrification, are more vulnerable to *NF1* dysfunction than others. The identified changes in brain organization are consistent with the patterns of cognitive dysfunction in the NF1 phenotype.

## Background

Brain development is dependent on a series of complex events including cellular proliferation, growth, differentiation and migration, programmed cell death and synaptic elimination. These events largely determine brain morphology [1]. Since brain structure is under significant genetic influence [2] it is important to use genetic models to understand its ontogeny.

Neurofibromatosis type 1 (NF1) is a good model in this respect, because it is a monogenic disorder caused by mutations in the *NF1* gene. The disorder has a prevalence of 1 in 3,500 [3] and is characterized by alterations in skin pigmentation (café-au-lait spots and skinfold freckling), increased tumor predisposition and learning deficits [4,5]. In the brain, neurofibromin, the protein product of the *NF1* gene, is expressed in both neurons and glial cells [6,7] and is required for neural development [8,9]. Loss of neurofibromin results in increased cellular growth [10], while it is also involved in learning and memory [11]. Thus, this disease provides a unique window into gene-brain-behavior relationships. The role of neurofibromin in cellular growth and its ubiquitous expression in the brain suggest that brain structure might be affected in patients. In fact, there is a high incidence of macrocephaly, optic gliomas and T2-weighted hyperintensities, commonly referred to as unidentified bright objects (UBOs) [12]. Nevertheless, the gross brain anatomy appears normal and it has been difficult to determine if brain structure is altered independent of focal lesions.

Recent advances in neuroimaging allow for an increasingly detailed delineation of developmental anomalies. Previous studies focusing on NF1 brain structure have examined the relative contributions of gray matter (GM) and white matter (WM) to increased brain size, with contradictory results [12]. The majority of studies point to macrocephaly being caused by increased WM [13,14] or a combination of WM and GM [15,16]. Only one study pointed to an increase in GM [17]. However, few reports attempted to identify regional abnormalities. Cutting *et al*. [15] focused on frontal and parietal lobe subdivisions and found they presented increased WM volume. Greenwood *et al*. [16] divided the brain into 16 parcellations and, besides a total increase in GM and WM volumes in patients with NF1, they reported increased GM in occipital and parietal regions and increased WM in anterior regions. Other brain structure anomalies observed included increased brainstem growth rate, suggestive of abnormal cell proliferation [18], smaller surface area and GM volume of the left planum temporale of NF1 boys compared with controls [19] and abnormal thalamic metabolic patterns observed with positron emission tomography [20] and magnetic resonance spectroscopy [21]. Furthermore, in a previous study from our laboratory, support vector machines were able to reveal the existence of brain structural differences in GM and WM tissue that could accurately discriminate individuals with NF1 from controls [22].

However, no previous study performed morphometric measurements of subcortical and cortical structures across the whole brain. Moreover, we extended our investigation beyond the volumetric dimension as alterations in neurofibromin expression might be differently reflected across the brain and manifest in distinct structures and morphological traits.

In this study, our aim was to provide a multidimensional morphometric analysis to clarify how brain structure is affected by NF1; to do this, we measured subcortical and cortical volumes, cortical thickness, cortical surface area and gyrification across the entire brain. The importance of assessing multiple morphometric traits is explained by the fact that they underlie distinctive evolutionary [23], developmental [24,25] and possible genetic [26] processes. A crucial point in our study was a careful matching of the control group, as several factors are known to influence brain morphology, including age [25], gender [27], intelligence [28,29] and handedness [30]. A number of previous studies focusing on structural alterations in NF1 were biased by gender [15] or included patients with brain tumors [16,17]. Moreover, the majority of studies lacked matching for intelligence quotient (IQ). In contrast, in the present study, patient and control groups did not show significant differences in age, gender, handedness or IQ.

The present multidimensional whole brain study is of an exploratory nature. However, given the previous reported imaging findings, the range of neuropsychological deficits observed in patients and the wide expression of neurofibromin in the brain we hypothesized that the pattern of alterations would not be limited to one brain region. Moreover, based on previous neuroimaging studies [15,16], we expected that the alterations observed will include frontal and parietal neocortical regions and at the subcortical level the corpus callosum [14,22,31-34].

## Methods

### Ethics statement

The study was conducted in accordance with the Declaration of Helsinki and all procedures were reviewed and approved by the Ethics Commissions of the Faculty of Medicine of the University of Coimbra and of the Children’s Hospital of Coimbra. Written informed consent was obtained from the parents/guardians of all participants. Children and adolescents gave written or oral informed consent.

### Subjects

The participants in this study belong to a larger cohort from our previous studies [22,35] and were selected so that the groups were matched for age, gender, IQ and handedness. We studied 14 patients with NF1 (mean age: 11.34 ± 2.51 SD; age range: 7.83 to 16.08 years; 6 males, 8 females) and 14 matched typically developing (TD) subjects (mean age: 11.89 ± 2.06 SD; age range: 7.83 to 15.33 years; 5 males, 9 females). All participants were right-handed.

Patients were recruited and diagnosed in collaboration with the Clinical Genetics Department of the Pediatric Hospital of Coimbra according to the NIH defined diagnostic criteria [36]. The TD group was recruited from a local school. Exclusion criteria for all participants were as follows: psychiatric disorder, neurological illness affecting brain function other than NF1, epilepsy, tumors or other clinically significant intracranial abnormality detected on magnetic resonance imaging (MRI). UBOs, areas of increased T_2_-weighted signal intensity on MRI with unknown etiology, commonly found in patients with NF1, were not considered exclusion criteria when present in patients. Additionally, we only included patients with intelligence quotient ≥90 in order to be able to match to typically developing children. On average, IQs reported in children with NF1 tend to be lower, with mean values around 90 (as reviewed by Ozonoff *et al*. [37]). Children prescribed with stimulant medication (methylphenidate) were not medicated on the day of MRI acquisition and neuropsychological assessment (3 NF1).

Neuropsychological assessment was performed using the Portuguese adapted version of the Wechsler Intelligence Scale for Children III (WISC-III) [38]. The demographic and neuropsychological characterization of both groups is shown in Table 1.

**Table 1:** Demographic and neuropsychological characterization

	**NF1 (n = 14)**	**TD (n = 14)**
Age	11.34 (2.51), range: 7.83 to 16.08	11.89 (2.06), range: 7.83 to 15.33
Gender (M:F)	6 M:8 F	5 M:9 F
Handedness (right:left)	14:0	14:0
FSIQ	104.36 (13.41), range: 90 to 126	110.00 (10.97), range: 90 to 124
VIQ	107.28 (11.38), range: 94 to 126	109.86 (11.90), range: 93 to 129
PIQ	99.43 (12.43), range: 80 to 119	109.07 (12.86), range: 87 to 136

Data are mean (SD).

NF1 = neurofibromatosis type 1; TD = typically developing; FSIQ = full-scale intelligence quotient; VIQ = verbal intelligence quotient;PIQ = performance intelligence quotient.

### MRI acquisition

Scanning was performed on a 3T Siemens TimTrio scanner, using a 12-channel birdcage head coil. In this study we analyzed acquired data with the following parameters: (i) two T_1_-weighted (T_1_w) magnetization-prepared rapid acquisition with gradient echo (MPRAGE) sequences, 1 × 1 × 1 mm voxel size, repetition time (TR) 2.3 s, echo time (TE) 2.98 ms, flip angle (FA) 9°, field of view (FOV) 256 × 256, 160 slices; (ii) a T_2_-weighted (T_2_w) fluid attenuated inversion recovery (FLAIR) sequence, 1 × 1 × 1 mm voxel size, TR 5 s, TE 2.98 ms, Inversion Time (TI) 1.8 s, FOV 250 × 250, 160 slices. FLAIR images were used to identify T_2_ hyperintensities. A neuroradiologist blinded to the participants’ clinical history observed the magnetic resonance structural scans and reported on the distribution and number of UBOs.

### MRI data analyses

Cortical reconstruction and volumetric segmentation were performed with the FreeSurfer image analysis suite (FreeSurfer v5.1.0, http://surfer.nmr.mgh.harvard.edu), as described in previous publications [39-41]. Briefly the processing included, motion correction, averaging of the two T_1_w images, registration to Talairach space, segmentation of the subcortical WM and deep GM volumetric structures [41,42], intensity normalization, tessellation of the GM/WM boundary, automated topology correction [43,44], and surface deformation following intensity gradients to optimally place the GM/WM and GM/cerebrospinal fluid (CSF) borders [39]. Image outputs were visually inspected and inaccuracies corrected when required. FreeSurfer provided correct segmentation and classification of subcortical structures in spite of the presence of UBOs in patients with NF1. UBOs were included as belonging to the structure they appeared on, as demonstrated in Additional file 1: Figure S1. Registration was performed to a spherical atlas [45]. Cortical thickness was calculated by computing the average shortest distance between the GM/WM and the GM/CSF surface [40]. Surface area maps of the GM/WM boundary were computed for each subject by calculating the area of every triangle in a cortical surface tessellation, as implemented in FreeSurfer. In anatomical regions of interest, surface area values were calculated by summing the triangular area tessellations included in the region. Local gyrification indices (LGIs) were computed using local measurements of gyrification over the whole cortical surface using the method developed by Schaer *et al*. [46]. The LGI reflects at each vertex the amount of cortical surface area than can be packed under a sphere with a fixed size.

Cortical volume, surface area, cortical thickness and the LGIs were estimated for 34 gyral regions per hemisphere by determining the mean values of each of these variables belonging to a gyral region of interest, as defined by the atlas developed by Desikan *et al*. [47], recently modified to include the insula as a region of interest. GM volume was estimated for subcortical structures. WM and GM brain volumes, WM and GM cerebellar volumes, WM and GM lobar volumes and total intracranial volumes (TIV) were also estimated for each participant. TIV was calculated using a validated method shown to be proportional to manually measured total intracranial volume [48]. Briefly, an atlas scaling factor is determined based on the transformation matrix of the atlas normalization for each individual subject. The atlas scaling factor is then used to scale the intracranial volume of the atlas to compute each subject TIV. Lobar metrics were extracted using FreeSurfer’s built-in lobe surface maps. The GM parcellation is based on the regions defined by the Desikan atlas [47] and the WM parcellation is performed as described in Salat *et al*. [49].

### Statistical analyses

Statistical analyses were performed with PAWS Statistics 18 (SPSS Inc., Chicago, IL, USA). First, we verified the normality assumption for the different parameters using the Shapiro-Wilk test. All data were normally distributed. Group differences in demographic and neuropsychological data were evaluated with independent samples t test, while a χ^2^ test was used for gender. Whole-brain volumetric analysis including overall GM and WM volumes, were assessed using independent samples t tests and also using analysis of covariance (ANCOVA) to control for TIV. Lobar and cerebellar WM and GM group differences were investigated using repeated measures ANCOVA with group (NF1 vs TD) as the between-subjects factor and hemispheres (left vs right) and brain regions (lobes/cerebellar WM/cerebellar GM) as the within-subjects factors and total intracranial volume (TIV) as covariate. Analyses of subcortical volumetric differences were assessed using multivariate ANCOVA with TIV as covariate. Cortical differences between groups were investigated using repeated measures ANCOVA with group (NF1 vs TD) as the between-subjects factor and hemispheres (left vs right) and brain regions (34 gyral regions) as the within-subjects factors and TIV as covariate. Follow-up tests were performed using multivariate ANCOVAs with TIV as covariate. Relationships between brain measurements and age were studied using Pearson’s correlations.

To control for type I errors we used the Benjamini and Hochberg [50] false discovery rate (FDR) method, which was applied per analysis. We set *q* = 0.05 or 0.1 (that is, 5% or 10% false positives). Multiple ROI-based analyses survived FDR control at *q* = 0.1 and several survived FDR control at *q* = 0.05. Although FDR at *q* = 0.1 can be considered a liberal correction for multiple comparisons, the exploratory nature of the present study should be taken into account.

## Results

### Demographic and neuropsychological

Demographic and neuropsychological data are described in Table 1. Children with NF1 and typically developing (TD) children did not differ significantly in age (t_(26)_ = −0.632, *P* = 0.533), IQ (t_(26)_ = −1.219, *P* = 0.234), verbal IQ (t_(26)_ = −0.584, *P* = 0.564), or gender (χ^2^ = 0.150, *P* = 0.699). Performance IQ was marginally significant (t_(26)_ = −2.016, *P* = 0.054), with larger scores observed in TD children.

One or more UBOs were present in 85.7% (12 out of 14) of the children with NF1 distributed as follows: 85.7% (n = 12) of the patients had UBOs in the globus pallidus, 21.4% (n = 3) in the thalami, 14.3% (n = 2) in the corpus callosum, 7.1% (n = 1) in the putamen, 14.3% (n = 2) in the cerebellum and 28.6% (n = 4) had UBOs in the WM. The frequency and distribution of UBOs is in accordance with published data [12]. None of the TD participants had UBOs. The number of UBOs was not correlated with total intracranial volume (r = 0.056, *P* = 0.850).

### Whole-brain analyses

Children with NF1 showed a 10% increase in total intracranial volume (TIV) as compared with the TD group (NF1: 1658 ± 127 cm^3^; TD: 1505 ± 138 cm^3^; t_(26)_ = 3.041, *P* = 0.005). This overall increase was more attributable to an increase in WM (20%) (NF1: 517 ± 61 cm^3^; TD: 428 ± 46 cm^3^; t_(26)_ = 4.340, *P* <0.001) than GM (8%), including cortical and subcortical GM (NF1: 789 ± 78 cm^3^; TD: 728 ± 74 cm^3^; t_(26)_ = 2.104, *P* = 0.045). After controlling for TIV, only WM remained different between groups (WM: F_(1,25)_ = 8.344, *P* = 0.008; GM: F_(1,25)_ = 1.044, *P* = 0.317), Table 2. Age was not significantly correlated with TIV for our subjects (r = 0.067, *P* = 0.736).

**Table 2:** Results of whole-brain analyses

	**NF1**	**TD**	***P *value**	**Controlling for TIV**		
				**NF1**	**TD**	***P *value**
Total cortical volume	584.60	544.42	0.094	555.35	573.68	0.238
Total cortical surface area	115099	107393	0.076	109580	112912	0.185
Mean cortical thickness	2.85	2.85	0.896	2.85	2.85	0.919
Mean local gyrification index	3.09	3.13	0.399	3.04	3.17	**0.007**
Gray matter volume	789.24	728.55	**0.045**	750.62	767.18	0.317
White matter volume	517.64	428.25	**<0.001**	490.43	455.47	**0.008**

Mean values after controlling for total intracranial volume represent estimate marginal means. Bold represents significant results (*P *<0.05). Volumes are expressed in cm^3^, surface in mm^2^, and thickness in mm.

NF1 = neurofibromatosis type 1; TD = typically developing; TIV = total intracranial volume.

**Figure 1:**
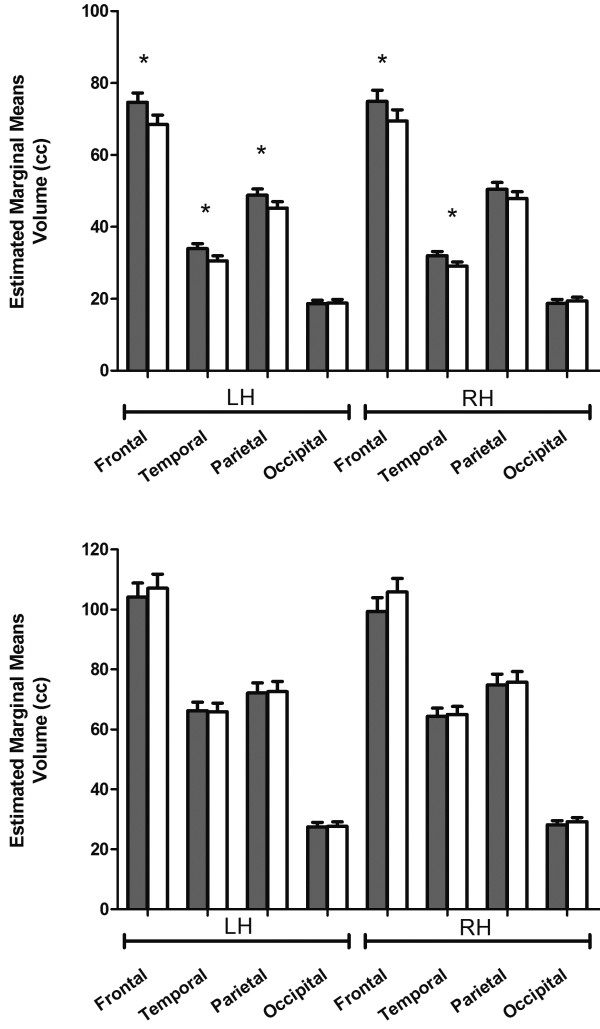
**Lobar volumes in patients with neurofibromatosis type 1 (NF1) (gray bars) and typically developing children (white bars). **Lobar volumes are displayed by hemisphere after controlling for total intracranial volume. **(A) **Lobar white matter. **(B) **Lobar gray matter. Graphs depict mean and SEM, *P <0.05. LH = left hemisphere; RH = right hemisphere.

We next examined overall volumetric differences at the lobar level. Results for WM are displayed in Figure 1A. Group differences in WM were reflected by a main effect of group (F_(1,25)_ = 8.028, *P* = 0.009), two-way interactions between hemisphere and group (F_(1,25)_ = 5.287, *P* = 0.030) and lobe and group (F_(3,75)_ = 6.758, *P* <0.001). Follow-up multivariate ANCOVA controlling for TIV, revealed that the left and right frontal lobes were significantly larger in patients (left frontal lobe: F_(1,25)_ = 10.456, *P* = 0.003; right frontal lobe: F_(1,25)_ = 5.786, *P* = 0.026), as well as left and right temporal lobes (left temporal lobe: F_(1,25)_ = 10.930, *P* = 0.003; right temporal lobe: F_(1,25)_ = 12.125, *P* = 0.002). However only the left hemisphere parietal lobe was significantly larger (left parietal lobe: F_(1,25)_ = 7.980, *P* = 0.009; right parietal lobe: F_(1,25)_ = 3.295, *P* = 0.081) and no differences were observed for the occipital lobe (left occipital lobe: F_(1,25)_ = 0.078, *P* = 0.782; right occipital lobe: F_(1,25)_ = 0.713, *P* = 0.406). Results for GM are displayed in Figure 1B. The lobar GM volumes included only cortical volumes and resulted in no significant effect of group and no interaction between group and lobe. Nonetheless, we found a significant two-way interaction between hemisphere and group (F_(1,25)_ = 8.468, *P* = 0.007).

At the cerebellar level, neither the cerebellar WM (left hemisphere: F_(1,25)_ = 0.042, *P* = 0.840; right hemisphere: F_(1,25)_ = 1.908, *P* = 0.179), nor the GM volumes (left hemisphere: F_(1,25)_ = 0.331, *P* = 0.570; right hemisphere: F_(1,25)_ = 0.612, *P* = 0.441) were statistically different between patients and TD children.

Total cortical measurements of volume, thickness and surface area were not different between patients with NF1 and TD children. However, mean LGI is significantly reduced in patients with NF1 when the increased intracranial volume in the patient group is taken into account (see Table 2).

### Subcortical regional brain volumes

**Figure 2:**
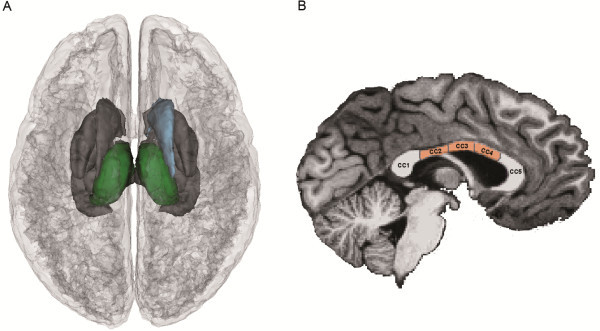
**Group differences in subcortical regional brain volumes: superior view (A) and sagittal view showing the divisions for the corpus callosum (B). **The larger volume in patients with neurofibromatosis type 1 (NF1) was observed in the left and right thalamus (green) and right caudate nucleus (blue) after controlling for total intracranial volume (TIV) and multiple comparisons using false discovery rate (FDR). In the corpus callosum, patients with NF1 showed larger volumes of the mid regions (mid-posterior, mid-anterior and central). CC = corpus callosum; CC1 = posterior; CC2 = mid-posterior; CC3 = central; CC4 = mid-anterior; CC5 = anterior.

Children with NF1 showed increased volumes of the thalami, the right caudate and the mid regions of the corpus callosum (mid-posterior, central, mid-anterior), Figure 2. These regions remained significant after correcting for multiple comparisons (FDR, *q* = 0.1), Table 3. Adding age or IQ as a covariates to the ANCOVA did not change the results.

**Table 3:** Results of analysis of covariance (ANCOVA) in subcortical regions

**Region**	**Hemisphere**	**Volume**		
		**F**	***P *value**	**DoD**
Thalamus	L	**6.106**	**0.021**^**a**^	**+**
	R	**9.415**	**0.005**^**a**^	**+**
Caudate	L	3.468	0.074	
	R	**6.861**	**0.015**^**a**^	**+**
Putamen	L	0.610	0.442	
	R	0.335	0.568	
Pallidum	L	0.344	0.563	
	R	0.023	0.880	
Hippocampus	L	3.942	0.058	
	R	0.108	0.746	
Amygdala	L	0.235	0.632	
	R	1.358	0.255	
Accumbens area	L	1.525	0.228	
	R	3.140	0.089	
Ventral diencephalon	L	4.242	0.050	
	R	2.748	0.110	
CC posterior		3.676	0.067	
CC mid-posterior		**5.587**	**0.026**^**a**^	**+**
CC central		**6.004**	**0.022**^**a**^	**+**
CC mid-anterior		**8.919**	**0.006**^**a**^	**+**
CC anterior		2.902	0.101	
Brainstem		2.563	0.122	

Bold represents significant results (*P* <0.05).

^a^Represents significant results after correction for multiple comparisons with FDR (*q* = 0.1). A plus sign indicate the direction (upwards) of the group difference, + = NF1 > typically developing subjects.

DoD = direction of difference; CC = corpus callosum; L = left; R = right; TIV = total intracranial volume.

### Cortical volume, cortical thickness and cortical surface area regional analyses

Cortical volume group differences, investigated using repeated measures ANCOVA with TIV as covariate, resulted in no main effect of group (F_(1,25)_ = 0.690, *P* = 0.414), but statistically significant two-way interactions between region and group (F_(33,825)_ = 1.651, *P* = 0.013) and hemisphere and group (F_(1,25)_ = 8.058, *P* = 0.009). Repeated measures ANCOVA of cortical thickness measurements resulted in no statistically significant effect of group (F_(1,25)_ = 1.084, *P* = 0.308), but a significant effect of brain region (F_(33,825)_ = 5.870, *P* <0.001), a two-way interaction between region and group (F_(33,825)_ = 1.522, *P* = 0.031) and a three-way interaction between hemisphere, region and group (F_(33,825)_ = 1.455, *P* = 0.049). The results for cortical surface area resemble those found for cortical volume and cortical thickness, with no significant effect of group (F_(1,25)_ = 0.378, *P* = 0.544). Nevertheless, we observed a significant two-way interaction between hemisphere and group (F_(1,25)_ = 6.061, *P* = 0.021). Significant interactions observed for the regional cortical analyses were explored in multivariate ANCOVAs. However, none of these differences survived correction for multiple comparisons (FDR, *q* = 0.1), Additional file 1: Table S1.

### Gyrification index

**Figure 3:**
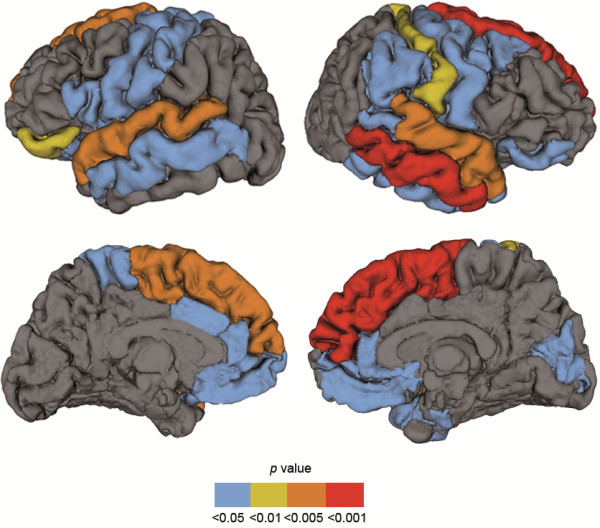
**Graphical illustration of significant reductions in gyrification in patients with neurofibromatosis type 1 (NF1). **This figure shows an overlay of F-test statistics (with *P *values indicated by the color bar) where patients with NF1 have lower local gyrification index than typically developing children. Between-group comparisons were performed controlling for intracranial volume. Blue color depicts gyri with significant group differences at *P *<0.05, not corrected; yellow *P* <0.01 and orange *P *<0.005, both surviving correction for multiple comparisons with FDR *q *= 0.1; red gyri *P *<0.001, survives correction for multiple comparisons with FDR *q *<0.05.

The gyrification index showed a significant effect of group (F_(1,25)_ = 10.400, *P* = 0.003), an effect of region (F_(33,825)_ = 3.263, *P* <0.001) and a two-way interaction between region and group (F_(33,825)_ = 2.591, *P* <0.001). Follow-up multivariate ANCOVA showed that patients with NF1 have reduced gyrification mainly in frontal and temporal lobar regions, but also in the insula, parietal, occipital and cingulate gyri, Table 4 and Figure 3. After correction for multiple comparisons several gyral regions remained statistically significant: left and right superior frontal, left and right superior temporal, right middle temporal and right transverse temporal (FDR, *q* = 0.05). The majority of gyral regions remained significant at FDR, *q* = 0.1 (Table 4). Adding age or IQ as covariates to the repeated measures ANCOVA and follow-up ANCOVAs did not change the results.

**Table 4:** Results of analysis of covariance (ANCOVA) for local gyrification index in gyral regions

**Gyral region**	**Hemisphere**	**Local gyrification index**		
		**F**	***P *value**	**DoD**
**Frontal lobe**				
Superior frontal	L	**12.315**	**0.002**^**b**^	**-**
	R	**11.938**	**0.002**^**b**^	**-**
Caudal middle frontal	L	1.074	0.310	
	R	**5.046**	**0.034**^**a**^	**-**
Rostral middle frontal	L	4.036	0.055	
	R	1.870	0.184	
Pars opercularis	L	**7.575**	**0.011**^**a**^	**-**
	R	4.071	0.054	
Pars triangularis	L	3.860	0.061	
	R	2.009	0.169	
Pars orbitalis	L	**8.324**	**0.008**^**a**^	**-**
	R	**4.869**	**0.037**^**a**^	**-**
Lateral orbitofrontal	L	**7.702**	**0.010**^**a**^	**-**
	R	1.797	0.192	
Medial orbitofrontal	L	**6.298**	**0.019**^**a**^	**-**
	R	**4.472**	**0.045**	**-**
Precentral	L	**5.693**	**0.025**^**a**^	**-**
	R	**6.354**	**0.018**^**a**^	**-**
Paracentral	L	**5.165**	**0.032**^**a**^	**-**
	R	2.847	0.104	
Frontal pole	L	**6.768**	**0.015**^**a**^	**-**
	R	1.366	0.253	
**Temporal lobe**				
Superior temporal	L	**11.052**	**0.003**^**b**^	**-**
	R	**24.242**	**<0.001**^**b**^	**-**
Middle temporal	L	**5.876**	**0.023**^**a**^	**-**
	R	**18.742**	**<0.001**^**b**^	**-**
Inferior temporal	L	0.312	0.582	
	R	**4.855**	**0.037**^**a**^	**-**
Entorhinal	L	2.504	0.126	
	R	**6.935**	**0.014**^**a**^	**-**
Fusiform	L	0.044	0.836	
	R	1.095	0.305	
Parahippocampal	L	0.013	0.911	
	R	0.529	0.474	
Temporal pole	L	3.399	0.077	
	R	**5.442**	**0.028**^**a**^	**-**
Transverse temporal	L	3.517	0.072	
	R	**14.003**	**0.001**^**b**^	**-**
Banks superior temporal sulcus	L	0.190	0.667	
	R	**5.689**	**0.025**^**a**^	**-**
**Parietal lobe**				
Superior parietal	L	1.640	0.212	
	R	0.125	0.727	
Inferior parietal	L	0.220	0.643	
	R	1.463	0.238	
Supramarginal	L	2.117	0.158	
	R	**5.233**	**0.031**^**a**^	**-**
Postcentral	L	**6.597**	**0.017**^**a**^	**-**
	R	**9.344**	**0.005**^**a**^	**-**
Precuneus	L	3.117	0.090	
	R	3.762	0.064	
**Occipital lobe**				
Lateral occipital	L	0.719	0.405	
	R	0.498	0.487	
Lingual	L	0.253	0.620	
	R	2.862	0.103	
Cuneus	L	3.589	0.070	
	R	**4.317**	**0.048**	**-**
Pericalcarine	L	1.915	0.179	
	R	**5.044**	**0.034**^**a**^	**-**
Rostral anterior cingulate	L	**4.848**	**0.037**^**a**^	**-**
	R	**5.487**	**0.027**^**a**^	**-**
Caudal anterior cingulate	L	**6.789**	**0.015**^**a**^	**-**
	R	3.213	0.085	
Posterior cingulate	L	2.052	0.164	
	R	3.165	0.087	
Isthmus cingulate	L	0.213	0.648	
	R	2.808	0.106	
Insula	L	**7.013**	**0.014**^**a**^	**-**
	R	**5.616**	**0.026**^**a**^	**-**

Bold represents significant results (*P *<0.05).

^a ^and ^b^Represent significant results after correction for multiple comparisons with false discovery rate (FDR), ^a^(*q* = 0.1) and ^b ^(*q *= 0.05 or 0.1). Minus indicates the direction of the group difference, - = NF1 < typically developing subjects.

DoD = direction of difference; L = left; R = right.

### Brain size and folding relationships

In humans, the volume of the brain and the area of its cortical surface are strongly correlated [51]. This relation is such that larger brains are normally accompanied by a higher increase in surface area than would be expected by mere scaling of the brain. Furthermore, increased folding is necessary for compactness of connections [52,53]. Indeed, when brain volume and surface area are plotted in a logarithmic scale the slope of the regression line obtained indicates a disproportionate expansion of the cerebral cortex in relation to brain volume, a result that is related to increased gyrification. Therefore we investigated whether this relationship was preserved in NF1.

**Figure 4:**
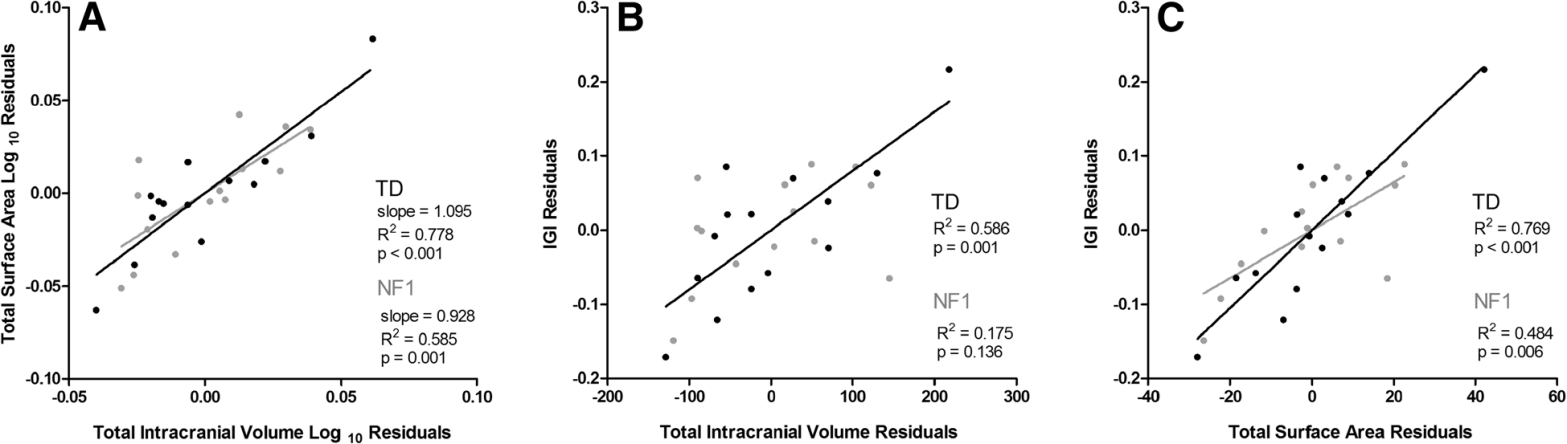
**Brain size and folding relationships in neurofibromatosis type 1 (NF1) (gray) and typically developing children (TD) (black), taking into account age and gender. (A) **Cortical surface area versus intracranial volume (log-log graph). **(B) **Local gyrification index (LGI) versus intracranial volume. **(C) **LGI versus surface area.

Figure 4A shows the relationship between cortical surface area and intracranial volume for patients with NF1 and TD children after controlling for age and gender, plotted in a log-log graph. TIV is correlated with surface area in both groups (NF1: r = 0.765, *P* = 0.004; TD: r = 0.882, *P* <0.001). The slope of the regression line is 0.928 for NF1 and 1.095 for TD children, higher than the value expected for an isometric scaling (2/3) as observed in the human brain [51].

To investigate whether the degree of cortical folding is related with measurements of TIV, surface area and cortical thickness, we performed partial correlations between these variables, controlling for age and gender. LGI was only positively correlated with TIV for TD subjects (NF1: r = 0.418, *P* = 0.176; TD: r = 0.765, *P* = 0.004), Figure 4B. The cortical surface area was correlated with LGI for both groups (NF1: r = 0.696, *P* = 0.012; TD: r = 0.877, *P* <0.001), Figure 4C; while LGI and cortical thickness were not correlated for any of the groups (not shown), consistent with prior studies [54-56].

## Discussion

In this study, we used computational methods to examine the impact of NF1, a single gene disorder, on multidimensional morphological brain traits. This is the first study to examine measurements of cortical thickness, cortical surface area and gyrification in this common neurodevelopmental genetic condition. We confirmed that children with NF1 have larger intracranial volumes than typically developing children (approximately 10%) and our findings indicate that this enlargement is mainly a result of increased WM volume. At the lobar level, our results indicate that patients with NF1 have larger WM volumes in the frontal lobe (consistent with prior studies [15,16,22]), temporal lobe (in agreement with voxel-based morphometry findings in a partly overlapping cohort [22]), and left parietal lobe. We did not find alterations in the occipital lobe. Our lobar data is in agreement with the notion that WM alterations are predominant in anterior brain regions. We also observed a parallel increase in GM volume in patients with NF1, a result attributable to larger subcortical rather than cortical volumes.

Measurements of subcortical regional volumes indicated that the thalami, the right caudate nucleus and the mid portions of the corpus callosum are significantly larger in patients with NF1 than TD children.

This study is the first, to the best of our knowledge, to report a volumetric alteration of the thalami in children with NF1. Moreover, we have previously identified this structure as belonging to a spatial pattern that significantly contributes to discriminate between brains from patients with NF1 and brains from controls [22]. Other studies have reported thalamic hypometabolism [20], T_1_ reduction [14] and abnormal choline content [21], possibly reflecting altered myelination.

The thalamus is a highly interactive structure with widespread connections to multiple cortical regions, and it provides selection and transformation of different sensory inputs to the cortex. The various nuclei of the thalamus are involved in the integration of sensory and motor information, memory and executive functions [57], competences in which NF1 patients show disabilities [4,58,59].

The right caudate nucleus was also found to be larger in patients and it was previously identified as a relevant structure to discriminate between NF1 and control subjects [22]. The caudate is a nucleus of the basal ganglia and therefore plays a role in sensorimotor coordination, while there is also evidence accumulating indicative of an involvement in goal-directed behavior [60]. It is widely connected with the frontal lobe and particularly with the dorsolateral prefrontal cortex [61,62], which is involved in working memory and executive function. A recent functional MRI (fMRI) study showed abnormal right caudate activation in NF1 in a spatial working memory task [63]. Moreover, Schrimsher *et al*. [64] observed that children with a greater degree of right to left caudate volume asymmetry show subclinical inattentive behaviors that define attention-deficit hyperactivity disorder (ADHD). Interestingly, there is a high incidence of ADHD in NF1 [4].

Finally, the enlargement observed in the midline portion of the corpus callosum corroborates previous reports from our group, in a partly overlapping cohort, using an independent method [22] and others [14,31-34]. Besides morphometric abnormalities, there is evidence of altered microstructure of the corpus callous in NF1 [65,66]. Behaviorally, higher volumes of corpus callosum were previously related to low IQ, impaired visuospatial and motor skills and learning problems in children with NF1 [17,34]. Interestingly, we observed that the volume of the corpus callosum remains abnormal even for normal IQs.

Unlike the specific observations concerning subcortical structures, examination of cortical measurements indicated that alterations in cortical volume, cortical surface area and cortical thickness are not significant. Gyrification index was the only changed measure in cortex.

One of the most evident features of human evolution is the increase in brain size [67]. In response to evolutionary demands, a high level of gyrification occurred in parallel with an increase in cortical GM in order to maximize the cortical surface while maintaining a smaller intracranial size [68]. The increase in folding in bigger brains seems to be necessary for the formation of efficient corticocortical connections in larger volumes [52,53]. This evolutionary trait appears disrupted in NF1, with an increase in brain size without a corresponding adaptive increase in folding. Accordingly, the correlation observed in TD children between LGI and intracranial volume is absent in NF1, indicating that patients with NF1 present lower gyrification indices than would be expected for their brain volume. The phylogenetic development of gyral and sulcal folds likely optimizes compaction of neuronal fibers while keeping neuronal signaling at an efficient transit time [52,53]. Given that abnormal cortical folding may reflect deficits in structural and, consequently, functional cortical connectivity, we will speculate on potential links between cortical folding abnormalities and the patterns of cognitive dysfunction that characterize the NF1 phenotype.

Concerning the cognitive phenotype related to cortical functions, executive functions have been reported to be impaired in children with NF1 [59,69], even when controlling for IQ [59]. Here, we observed lower LGI in several frontal lobe regions that could underlie executive impairments, namely the superior frontal gyri [70], right frontal pole [71]and orbitofrontal regions [72,73]. Moreover, the anterior cingulate cortex was also found to have lower LGI values in NF1 and it plays a role in attention and error detection [74]. Interestingly, we have previously found an abnormal activity pattern in this region using an overlapping cohort of subjects [35], possibly related with a deficit in default-mode network function.

Patients with NF1 have deficits in expressive and receptive language, vocabulary and phonologic awareness [58]. Previously, a correlation has been reported between verbal skills in NF1 and the inferior frontal gyrus morphology, such that individuals with ‘typical’ gyral patterns in the right hemisphere performed worse across language measures than those showing ‘atypical’ gyrus [75]. Here, we observed a deficit in gyrification in regions involved with language functions, namely in regions belonging to the Broca’s complex (pars opercularis, pars orbitalis, pars triangularis) [76,77], superior temporal gyrus and middle temporal gyrus [78,79] and supramarginal gyrus [80,81]. Moreover, right transverse temporal gyrus, known as the Heschl’s gyrus, also presented lower LGIs in patients. Abnormalities in the inferior frontal gyrus and Heschl’s gyrus were associated with performance across language and neuropsychological measures in individuals with NF1 [75].

Motor deficits for both simple and complex motor tasks have been reported in NF1 [58,82,83] consistent with the observed bilateral deficits in LGI in both precentral and paracentral gyri, underlying motor functions [84].

In spite of our identification of altered patterns of gyrification in regions underlying cognitive deficits typically observed in children with NF1, this was not the case for the posterior parietal lobe. This is rather surprising given that visuospatial deficits are considered a hallmark of the NF1 cognitive phenotype. This might be explained by the suggestion that other brain regions such as frontoexecutive regions contribute to the pattern of impairment observed in specific visuospatial tests (that is, judgment of line orientation test).

In agreement with the notion of impaired low-level visual processing in NF1 [35,85], we observed lower LGI in the right cuneus and pericalcarine regions.

It is estimated that around 30% of phenotypic variance in gyrification is attributed to genetic variation [86]. Therefore, the observed reductions in LGI point towards early abnormalities in control over neuronal migration or proliferation. Deeper fissures develop earlier and are more strongly influenced by genetic processes and thus might be less susceptible to environmental perturbations. Insular sulci and the central sulcus are among the first macroscopical structures identified on the human fetal brain [87,88], and both the insula and the gyri surrounding the central sulcus presented less gyrification in NF1 than TD individuals.

The mechanisms that drive cortical folding remain poorly understood and there are different possible explanations for the observed abnormalities of cortical gyrification in NF1. The two most widely accepted hypothesis to explain gyrification are: (1) folding is caused by differential growth of the cortex and (2) folding is caused by mechanical tension generated in axons. The first model proposes that differential growth rates of cortical layers directly affect the degree of cortical convolutions [89]. An alternate theory to this hypothesis suggests that changes in subcortical connections can lead to altered cortical folding patterns without changing the area of the cortical surface [90]. The second model, based on tension-based cortical morphogenesis, proposes that the mechanical tension along axons is the driving force for cortical folding [91]. In line with this theory, we observed a significant increase in WM in the anterior NF1 brain accompanied by an enlargement of the corpus callosum. Moreover, diffusion tensor imaging studies showed altered microstructure in several white matter regions [66]. Both models are not necessarily contradictory and could jointly explain the gyral abnormalities in NF1.

The main limitation of the present study is that we investigated a large age range and we were limited to a relatively small number of participants. In that sense, longitudinal studies are clearly warranted to disentangle the complex genetic and non-genetic influences that contribute to the neuroanatomic and cognitive abnormalities in NF1.

## Conclusions

We found that the overall increase in intracranial volume in patients with NF1 can be explained by an enlarged WM volume in anterior brain regions and subcortical GM volumes, with a disproportionate increase in the volumes of the thalami, right caudate nucleus and corpus callosum. At the cortical level we observed abnormalities of gyrification, which could reflect developmental abnormalities in both cortical architecture and corticocortical connectivity. This newly identified pattern of gyral malformation required quantitative tools such as LGI to be detected. Given the emphasis on a careful matching between patients and TD children we believe that the brain alterations observed in here are shaped primarily by genetically programmed anomalous neurodevelopment. The fact that neuroanatomic abnormalities in patients with NF1 are localized to particular brain regions adds to the growing body of evidence that specific genes independently control the morphometry of specific cytoarchitectonic areas.

## Competing interests

The authors have no financial or non-financial competing interests to declare.

## Authors’ contributions

IRV, MJR, ES and MCB conceptualized and designed the study. IRV collected and analyzed the data. All authors contributed to interpreting results and writing the manuscript. All authors read and approved the final manuscript.

## Supplementary Material

Additional file 1 Supplementary material. Click here for file
