# Supplementary material for: Gyrification, cortical and subcortical morphometry in neurofibromatosis type 1: an uneven profile of developmental abnormalities
Source: J Neurodev Disord. 2013 Feb 13;5(1):3. doi: 10.1186/1866-1955-5-3 (PMC3599251; doi:10.1186/1866-1955-5-3)
Supplement: Additional file 1 — Supplementary material. [file 1866-1955-5-3-S1.docx]

**
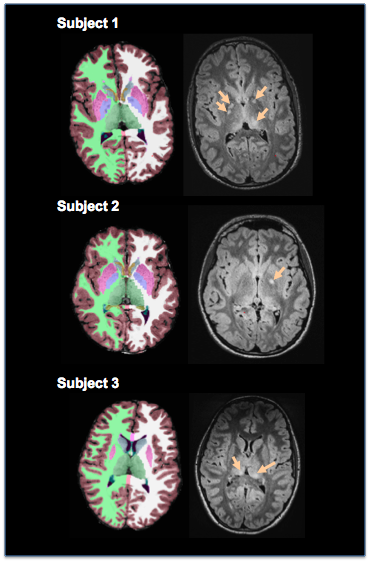
Supplementary Material**

**Supplementary Figure 1. FreeSurfer segmentation in three patients with NF1 with unidentified bright objects (UBOs).** UBOs positions are indicated with an orange arrow in a T2-weighted image (left side) corresponding to the same slice showing FreeSurfer segmentation (right side).

**Supplementary Table 1.** Results of ANCOVA analyses of cortical volume, cortical thickness and cortical surface area

| Gyral region | Hemisphere | Cortical volume | |  | Cortical thickness | |  | Cortical surface area | |  |
| --- | --- | --- | --- | --- | --- | --- | --- | --- | --- | --- |
|  |  | F | *p* |  | F | *p* |  | F | *p* |  |
| Frontal lobe | | | | | | | | | | |
| Superior frontal | L | 2.172 | 0.153 |  | 0.002 | 0.965 |  | 1.273 | 0.270 |  |
|  | R | 3.075 | 0.092 |  | 0.154 | 0.698 |  | 1.703 | 0.204 |  |
| Caudal middle frontal | L | 0.493 | 0.489 |  | 0.210 | 0.650 |  | 0.138 | 0.713 |  |
|  | R | **9.829** | **0.004** | ↓ | 1.170 | 0.290 |  | **4.490** | **0.044** | ↓ |
| Rostral middle frontal | L | 0.698 | 0.411 |  | 0.058 | 0.812 |  | 0.443 | 0.512 |  |
|  | R | 1.942 | 0.176 |  | **4.385** | **0.047** | ↓ | 0.418 | 0.524 |  |
| Pars opercularis | L | 0.460 | 0.504 |  | 0.414 | 0.526 |  | 0.800 | 0.380 |  |
|  | R | 0.279 | 0.602 |  | 0.108 | 0.745 |  | 0.192 | 0.665 |  |
| Pars triangularis | L | 0.122 | 0.729 |  | 2.352 | 0.138 |  | 0.217 | 0.645 |  |
|  | R | 0.497 | 0.487 |  | 0.016 | 0.901 |  | 0.335 | 0.568 |  |
| Pars orbitalis | L | 1.509 | 0.231 |  | 2.560 | 0.122 |  | 0.748 | 0.395 |  |
|  | R | 0.008 | 0.929 |  | 0.150 | 0.702 |  | 0.760 | 0.392 |  |
| Lateral orbitofrontal | L | 0.036 | 0.851 |  | 0.001 | 0.976 |  | 0.437 | 0.515 |  |
|  | R | 0.143 | 0.709 |  | 0.861 | 0.362 |  | 0.185 | 0.671 |  |
| Medial orbitofrontal | L | 0.184 | 0.672 |  | 0.455 | 0.506 |  | 0.095 | 0.760 |  |
|  | R | 3.166 | 0.087 |  | 0.303 | 0.587 |  | 0.084 | 0.774 |  |
| Precentral | L | 1.354 | 0.256 |  | 0.192 | 0.665 |  | 0.008 | 0.932 |  |
|  | R | 0.001 | 0.981 |  | 3.371 | 0.078 |  | 0.086 | 0.772 |  |
| Paracentral | L | 0.233 | 0.633 |  | 1.299 | 0.265 |  | 1.749 | 0.198 |  |
|  | R | 0.021 | 0.885 |  | 0.002 | 0.963 |  | 0.005 | 0.946 |  |
| Frontal pole | L | 2.172 | 0.153 |  | 0.035 | 0.853 |  | 0.458 | 0.505 |  |
|  | R | 3.075 | 0.092 |  | 2.298 | 0.142 |  | 1.095 | 0.305 |  |
| Temporal lobe |  |  |  |  |  |  |  |  |  |  |
| Superior temporal | L | 0.449 | 0.509 |  | **5.624** | **0.026** | ↑ | 0.383 | 0.542 |  |
|  | R | 0.001 | 0.975 |  | **5.738** | **0.024** | ↑ | 0.041 | 0.841 |  |
| Middle temporal | L | 0.780 | 0.385 |  | 0.940 | 0.341 |  | 0.003 | 0.959 |  |
|  | R | 1.190 | 0.286 |  | 0.673 | 0.420 |  | 0.000 | 0.998 |  |
| Inferior temporal | L | 0.275 | 0.605 |  | 0.357 | 0.556 |  | 1.776 | 0.195 |  |
|  | R | 0.043 | 0.837 |  | 0.030 | 0.863 |  | 0.524 | 0.476 |  |
| Ethorhinal | L | 0.015 | 0.904 |  | 0.898 | 0.352 |  | 0.000 | 0.983 |  |
|  | R | 1.135 | 0.297 |  | 0.079 | 0.781 |  | 1.210 | 0.282 |  |
| Fusiform | L | 0.030 | 0.863 |  | 0.239 | 0.629 |  | 1.623 | 0.214 |  |
|  | R | 0.060 | 0.809 |  | 0.256 | 0.617 |  | 0.020 | 0.890 |  |
| Parahippocampal | L | 0.115 | 0.737 |  | 0.373 | 0.547 |  | 0.401 | 0.532 |  |
|  | R | 0.017 | 0.896 |  | 2.184 | 0.152 |  | 0.173 | 0.681 |  |
| Temporal pole | L | 0.190 | 0.667 |  | 0.553 | 0.464 |  | 0.296 | 0.591 |  |
|  | R | 3.074 | 0.092 |  | 0.294 | 0.593 |  | 1.809 | 0.191 |  |
| Transverse temporal | L | 0.834 | 0.370 |  | 2.618 | 0.118 |  | 1.789 | 0.193 |  |
|  | R | 0.083 | 0.776 |  | **6.249** | **0.019** | ↑ | 0.875 | 0.358 |  |
| Banks superior temporal sulcus | L | 3.818 | 0.062 |  | 0.509 | 0.482 |  | 2.552 | 0.123 |  |
|  | R | 2.335 | 0.139 |  | 3.140 | 0.089 |  | 2.824 | 0.105 |  |
| Parietal lobe |  |  |  |  |  |  |  |  |  |  |
| Superior parietal | L | 1.102 | 0.304 |  | 2.408 | 0.133 |  | 0.986 | 0.330 |  |
|  | R | 2.192 | 0.151 |  | 1.347 | 0.257 |  | 2.442 | 0.131 |  |
| Inferior parietal | L | 0.560 | 0.461 |  | 2.894 | 0.101 |  | 0.535 | 0.471 |  |
|  | R | 0.951 | 0.339 |  | 0.149 | 0.703 |  | 1.079 | 0.309 |  |
| Supramarginal | L | 0.874 | 0.359 |  | 0.715 | 0.406 |  | 0.611 | 0.442 |  |
|  | R | 0.334 | 0.568 |  | 0.802 | 0.379 |  | 0.037 | 0.850 |  |
| Postcentral | L | 0.496 | 0.488 |  | **4.598** | **0.042** | ↑ | 0.019 | 0.892 |  |
|  | R | 0.030 | 0.865 |  | 1.292 | 0.266 |  | 1.148 | 0.294 |  |
| Precuneus | L | 0.587 | 0.451 |  | 0.072 | 0.790 |  | 0.136 | 0.716 |  |
|  | R | 0.993 | 0.329 |  | 0.207 | 0.653 |  | 0.101 | 0.753 |  |
| Occipital lobe |  |  |  |  |  |  |  |  |  |  |
| Lateral occipital | L | 2.160 | 0.154 |  | **8.786** | **0.007** | ↑ | 0.375 | 0.546 |  |
|  | R | 0.150 | 0.702 |  | 2.879 | 0.102 |  | 0.713 | 0.406 |  |
| Lingual | L | **4.558** | **0.043** | ↓ | 0.002 | 0.963 |  | **6.174** | **0.020** | ↓ |
|  | R | 3.357 | 0.079 |  | 0.796 | 0.381 |  | **4.380** | **0.047** | ↓ |
| Cuneus | L | 0.075 | 0.786 |  | 3.532 | 0.072 |  | 0.022 | 0.883 |  |
|  | R | 0.005 | 0.947 |  | **7.230** | **0.013** | ↑ | 0.933 | 0.343 |  |
| Pericalcarine | L | 1.537 | 0.227 |  | 1.257 | 0.273 |  | 2.232 | 0.148 |  |
|  | R | 1.024 | 0.321 |  | **4.949** | **0.035** | ↑ | 3.477 | 0.074 |  |
| Rostral anterior cingulate | L | 0.512 | 0.481 |  | 0.121 | 0.731 |  | 0.140 | 0.712 |  |
|  | R | 3.073 | 0.092 |  | 0.033 | 0.857 |  | **5.575** | **0.026** | ↑ |
| Caudal anterior cingulate | L | 0.010 | 0.920 |  | 0.074 | 0.788 |  | 0.014 | 0.906 |  |
|  | R | **4.913** | **0.036** | ↑ | 1.217 | 0.280 |  | **5.098** | **0.033** | ↑ |
| Posterior cingulate | L | 0.671 | 0.421 |  | 1.064 | 0.312 |  | 0.008 | 0.929 |  |
|  | R | 2.582 | 0.121 |  | 2.060 | 0.164 |  | 1.124 | 0.299 |  |
| Isthmus cingulate | L | 1.282 | 0.268 |  | 0.872 | 0.359 |  | 1.144 | 0.295 |  |
|  | R | 1.703 | 0.204 |  | **6.951** | **0.014** | ↑ | **4.532** | **0.043** | ↓ |
| Insula | L | 3.240 | 0.084 |  | 2.041 | 0.165 |  | 2.867 | 0.103 |  |
|  | R | **5.331** | **0.029** | ↓ | 0.117 | 0.735 |  | **5.140** | **0.032** | ↓ |

Bold font represents significant results (*p* < 0.05). ^a^ Represents significant results after correction for multiple comparisons with FDR (*q* = 0.05 or 0.1) and ^b^ after with FDR (*q* = 0.1). Arrows indicate the direction of the group difference, ↓ = NF1 < Typically developing subjects. L = left; R = right
